# Machine learning identifies right index finger tenderness as key signal of DAS28-CRP based psoriatic arthritis activity

**DOI:** 10.1038/s41598-023-49574-4

**Published:** 2023-12-19

**Authors:** Samuel Rischke, Sorwe Mojtahed Poor, Robert Gurke, Lisa Hahnefeld, Michaela Köhm, Alfred Ultsch, Gerd Geisslinger, Frank Behrens, Jörn Lötsch

**Affiliations:** 1 Institute of Clinical Pharmacology, Goethe-University, Theodor-Stern-Kai 7, 60590 Frankfurt Am Main, Germany; 2 Fraunhofer Institute for Translational Medicine and Pharmacology ITMP, Theodor-Stern-Kai 7, 60596 Frankfurt Am Main, Germany; 3 Fraunhofer Cluster of Excellence Immune Mediated Diseases CIMD, Theodor-Stern-Kai 7, 60596 Frankfurt Am Main, Germany; 4 Department of Rheumatology, Goethe University Frankfurt, University Hospital, Theodor-Stern-Kai 7, 60590 Frankfurt Am Main, Germany; 5 DataBionics Research Group, University of Marburg, Hans-Meerwein-Straße, 35032 Marburg, Germany

**Keywords:** Rheumatic diseases, Autoimmune diseases, Data processing, Machine learning

## Abstract

Psoriatic arthritis (PsA) is a chronic inflammatory systemic disease whose activity is often assessed using the Disease Activity Score 28 (DAS28-CRP). The present study was designed to investigate the significance of individual components within the score for PsA activity. A cohort of 80 PsA patients (44 women and 36 men, aged 56.3 ± 12 years) with a range of disease activity from remission to moderate was analyzed using unsupervised and supervised methods applied to the DAS28-CRP components. Machine learning-based permutation importance identified tenderness in the metacarpophalangeal joint of the right index finger as the most informative item of the DAS28-CRP for PsA activity staging. This symptom alone allowed a machine learned (random forests) classifier to identify PsA remission with 67% balanced accuracy in new cases. Projection of the DAS28-CRP data onto an emergent self-organizing map of artificial neurons identified outliers, which following augmentation of group sizes by emergent self-organizing maps based generative artificial intelligence (AI) could be defined as subgroups particularly characterized by either tenderness or swelling of specific joints. AI-assisted re-evaluation of the DAS28-CRP for PsA has narrowed the score items to a most relevant symptom, and generative AI has been useful for identifying and characterizing small subgroups of patients whose symptom patterns differ from the majority. These findings represent an important step toward precision medicine that can address outliers.

## Introduction

Psoriatic arthritis (PsA) is a chronic inflammatory systemic disease that affects approximately 20–30% of psoriasis patients and presents with skin, nail, and musculoskeletal manifestations^1,2^. Due to the complex pathogenesis and heterogeneous expression of inflammation in peripheral and axial joints, entheses, and tendons, differential diagnosis and subsequent monitoring of PsA can be challenging. The disease activity of peripheral PsA is often evaluated using the Disease Activity Score 28 (DAS-28 CRP), which measures tender and swollen joint counts, blood concentration of C-reactive protein, and self-rated global health^3,4^. Although originally developed for rheumatoid arthritis, the DAS28-CRP has been commonly used for PsA.

Studies of drug effects on PsA have used DAS28-CRP outcomes in approximately one-tenth of the published studies, as identified through a search of the PubMed database on April 26, 2023, at https://pubmed.ncbi.nlm.nih.gov for “(((((psoriatic arthritis) AND (clinic* or patient)) AND (drug or pharmacological*)) AND (therapy)) NOT (review[publication type])”, which provided 3,450 hits, while adding DAS28 as “AND (DAS28-CRP OR das28* OR (das 28) OR das-28 OR Disease Activity Score 28))” yielded 280 hits. A more specific search for interventional or observational clinical trials in PsA in the clinicaltrials.gov database at https://clinicaltrials.gov, using "DAS28 CRP" as an additional keyword, yielded 29 trials that based at least one of their outcome measures on DAS28-CRP (Table 1). The present clinical investigation primarily focused on the DAS28-CRP score for evaluating PsA activity. However, it is worth noting that several alternative scoring systems have been proposed to assess PsA activity, taking into account specific disease characteristics (for a comprehensive review, see^4^). These alternatives encompass criteria such as nail involvement (e.g., Nail Assessment Psoriasis Severity Index—NAPSI), joint inflammation (e.g., Swollen Joint Count—SJC 66), spondylitis (e.g., Maastricht Ankylosing Spondylitis Enthesis Score—MASES), and quality of life (e.g., Dermatological Life Quality Index—DLQI). Additionally, some scores provide a comprehensive evaluation of PsA, such as the Disease Activity Psoriatic Arthritis Score (DAPSA), or the Psoriatic Activity Joint Index (PSARC). It should be noted that previous work has found a high correlation between DAS28-CRP and PsA-specific indices such as DAPSA^5^.

**Table 1:** Completed or ongoing clinical trials in PsA according to a search for interventional or observational trials in PsA in the clinicaltrials.gov database at https://clinicaltrials.gov, using "DAS28-”RP" as keyword, conducted on April 28, 2023.

Type	Scope	Phase	Count	Drug targets
Interventional	Drug Trial	II	5	*IL12/23-antibodies (1), IL23-antibodies (1), TYK-inhibitors (1), MK2-inhibitors (1),* *PDE4-inhibitors (1)*
		III	14	*IL17A-antibodies (9), IL23-antibodies (2),* *TNFα-inhibitors (1), JAK-inhibitors (3),* *PDE4-inhibitors (1)*
		IV	4	*IL17A-antibodies (2), TNFα-inhibitors (1),* *PDE4-inhibitors (1),* *tRNA-synthase-inhibitors (1)*
	Medicinal Product Trial		1	
	Clinical Research		2	
Observational	Clinical Research		3	

The trials listed included DAS28-CRP among their predefined clinical endpoints, such as "DAS28-CRP change from baseline", and a categorization of PsA activity, such as "percentage of subjects categorized as low or remission".

Given the established use of DAS28-CRP for evaluating PsA activity, this study is dedicated to an evaluation its application to PsA activity, with a specific emphasis on understanding the significance of individual components within the score for this purpose within predominantly peripheral PsA. A machine learning workflow was employed to distill the most informative components for assessing polyarthritic PsA activity based on arthritic involvement. The algorithm was specifically trained to detect remission through the assessment of arthritis severity, and the data structure underwent a thorough analysis to unveil insights through machine learning-based data structure detection^6^.

## Methods

### Patients and study design

This was a cross-sectional study enrolling patients with rheumatic diseases. The study was conducted in accordance with the Declaration of Helsinki on Biomedical Research Involving Human Subjects and was approved by the Ethics Committee of the Medical Faculty of the Goethe-University, Frankfurt am Main, Germany (approval number 19-492_5). Informed written consent was obtained from each of the participants.

Patients were enrolled between May 10, 2020 and October 7, 2021. Inclusion criteria included a minimum age of 18 years and a diagnosis of arthritis, collagenosis (specifically systemic lupus erythematosus), or vasculitis. The available data set originally included n = 117 patients with rheumatic diseases, including psoriatic arthritis (n = 80), systemic lupus erythematosus (n = 19), and various forms of vasculitis (n = 18), of which n = 2 were granulomatosis with polyangiitis (GPA). The remaining n = 45 subjects were healthy controls. The data analyzed were collected at the first visit after enrollment. The present analyses focused on the clinical and therapeutic characteristics of the n = 80 PsA patients (44 women, 36 men, age 56.3 ± 12.0 years (mean ± standard deviation; range 25—79 years; body mass index, BMI = 27.9 ± 4.8 kg/m^2^). The initial diagnosis of PsA at the first visit was 16.4 ± 12 years on average; this information could not be obtained for 22 of the patients.

### Data analysis

**Figure 1:**
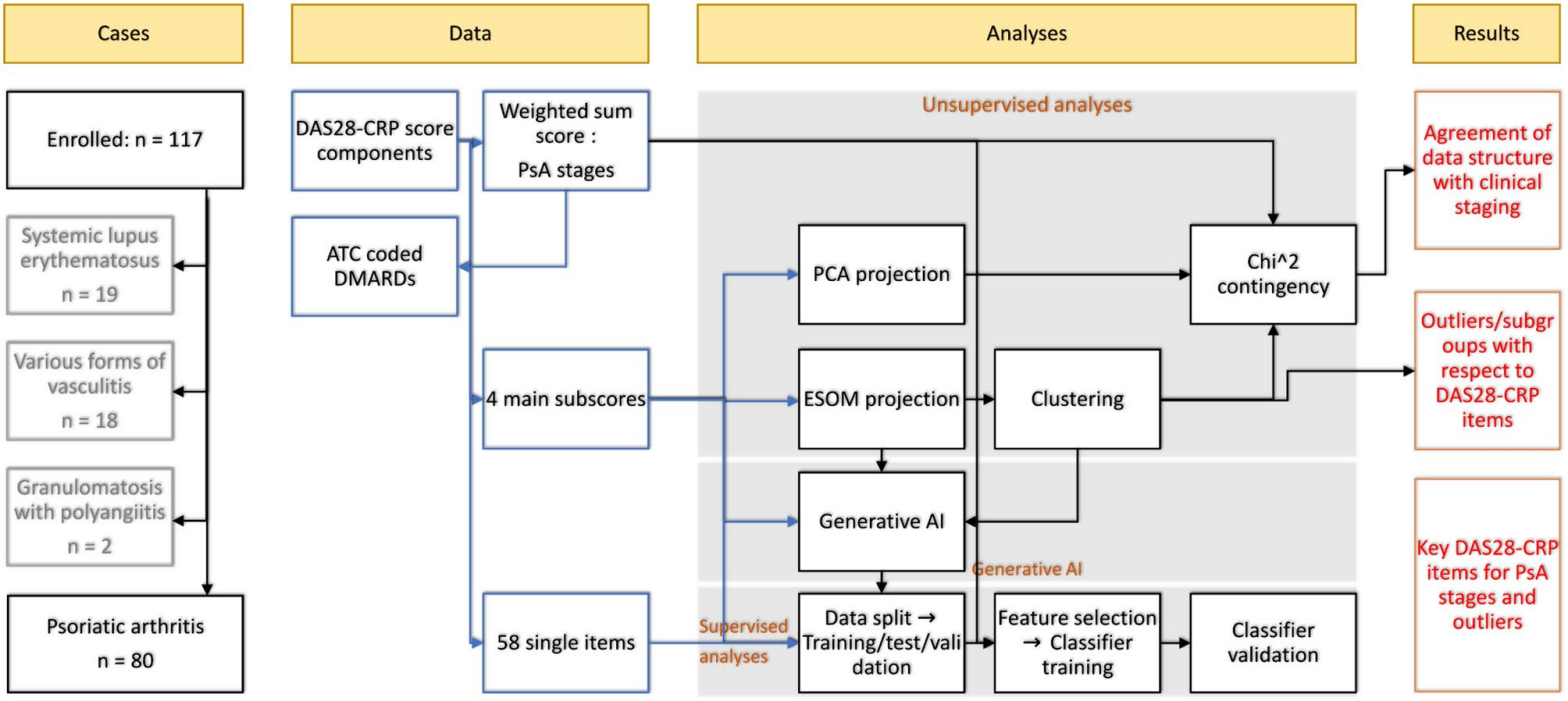
Flowchart of the study cases and data analysis. The data analysis was designed to use unsupervised methods to identify structures in the clinical data that supported the prior classification into PsA stages (remission or not, or remission, low or moderate), and to use generative AI and supervised methods to extract the most relevant items among the DAS28-CRP score items for prior classification and subgroups (clusters) or outliers detected in the DAS28-CRP pattern following data projection of a two-dimensional grid of artificial neurons (ESOM/U-matrix). The figure was created using Microsoft PowerPoint® (Redmond, WA, USA) on Microsoft Windows 11 running in a virtual machine powered by VirtualBox 7.0.6 (Oracle Corporation, Austin, TX, USA) as guest on Linux, and then further modified with the free vector graphics editor “Inkscape” (version 1.2.2 for Linux, https://inkscape.org/).

Data analysis was designed to identify structures in the clinical and drug-treatment related data that supported the prior classification of PsA patients by disease activity levels using unsupervised analyses for structure detection, and to extract elements of the Disease Activity Score-28 with CRP (DAS28-CRP) that were most informative for disease activity classification, using supervised methods of feature selection and classification. In addition, the association between drug therapy and the severity of the four components of the DAS28-CRP (number of tender joints, number of swollen joints, C-reactive protein (CRP), and patient reported global health status in a visual analogue scale) was examined. An overview of the data analysis strategy is presented in Fig. 1.

The software coding was done in the R language^7^ using the R software package^8^, version 4.3 for Linux, freely available from the Comprehensive R Archive Network (CRAN) at https://CRAN.R-project.org/, and in the Python language^9^ using Python version 3.8.16 for Linux, freely available from https://www.python.org (accessed 24 April 2023) and running in the Anaconda data science environment (Anaconda Inc., Austin, TX, USA; https://www.anaconda.com). The analyses were performed on an AMD Ryzen Threadripper 3970X (Advanced Micro Devices, Inc., Santa Clara, CA, USA) desktop computer running Ubuntu Linux 22.04.2 LTS (Canonical, London, UK).

#### Unsupervised analyses of DAS28-CRP items

Unsupervised analyses to identify structure in the DAS28-CRP data consisted of projections of its four components (number of tender joints, number of swollen joints (out of 28 pre-defined distal joints), C-reactive protein (CRP), and global health status) on the two-dimensional ${\mathbb{R}}^{2}$ plane, using classical principal component analysis (PCA)^10,11^ of the z-standardized data. This was done with the R library “FactoMineR”^12^.

Independently, the DAS28-CRP data, scaled to the range [0,…,100] were projected onto an emergent self-organizing (ESOM) map of artificial neurons as self-organizing maps (SOM) of artificial neurons^13^. In their special form of ESOM, i.e., emergent SOM^14,15^), the maps consisted of 4,000 neurons arranged on a two-dimensional toroidal grid with 50 rows and 80 columns^14,16^). After training the artificial network in 20 epochs using learning rates from 0.3 to 0.05 and a Gaussian neighborhood function, the distances between the neurons representing a prototype were calculated using the so-called U-matrix^17,18^, where the "height" represents the average high-dimensional distance of a prototype with respect to all immediately neighboring prototypes. The corresponding visualization technique uses a topographic map including coloring to enhance the emergence of a cluster structure. The latter allowed direct comparison of the subgroup structure detected in the data with the prior classification according to PsA activity stages using standard χ^2^ statistics^19^ as implemented in the R library “vcd”^20^. All ESOM-based analyses were done using our R library “Umatrix”^15^.

#### Supervised analyses of DAS28-CRP items

After verifying that the structure in the four DAS28-CRP main items was consistent with the prior PsA activity subgrouping and/or contained further structure, supervised analyses were performed to assess which of the 58 singular DAS28-CRP components down to the individual joint level were the main players determining this structure. Due to the small group size of cases with moderate PsA activity (n = 5, see also Results section), binary classifiers were trained to distinguish PsA in remission from active cases, the latter being combined cases with low or moderate disease activity. Given the heterogeneous data scaling in the DAS28-CRP, including interval scaled and binomial variables, random forests^21,22^ was selected as a widely used classifier with good performance on tabular numerical data, where it has been shown to be comparable to or even superior to other methods^23^, ranging from logistic regression^24^ to deep learning neural networks^25,26^. Its suitability for the DAS28-CRP variables is because no complicated variable transformations or scaling are required, as is common with competitors. Random forests algorithm was implemented in Python. The main packages used were the numerical Python package "numpy"^27^, "pandas"^28^, fundamental algorithms for scientific computing in Python “SciPy”^29^ and "scikit-learn"^30^.

Before supervised classifier learning was applied, the dataset was split into a training/test subset containing 66.67% of the cases and a validation subset containing the remaining 33.33%. The latter was not touched for feature selection and classifier training. It was used only for performance measures of the final classifier. Hyperparameter tuning was done using the "Optuna" hyperparameter optimization framework, installable from https://optuna.org, which provides Bayesian optimization tools that were used in 200 iterations each with fivefold cross-validation. After tuning, random forest classifiers were trained and the generic permutation feature importance provided in the "permutation_importance" method of the "sklearn.inspection" package was calculated, setting the number of permutations to n = 50 repeats. The mean importance measure calculated for each DAS28-CRP item was subjected to a computed ABC (cABC) analysis^31^ to obtain the subset of the most relevant score items. Of note, the cABC method is an item categorization technique that divides a set of positive numeric data into three disjoint subsets, labeled "A" to "C". Subset "A" contains the "important few", which are retained as "reduced" feature sets, while subset "C" contains the "trivial many"^32^. The Python implementation is available as our package "cABCanalysis"^33^.

The classifier was then trained with the full and reduced feature sets in a 4 × 25 nested cross-validation scenario^34^ with Monte-Carlo resampling^35^, using subsets of a 66.67%-training/test sample separated from the full original dataset prior to feature selection and classifier tuning. Hyperparameter tuning was repeated for each data subset. The trained classifier was then applied to random subsets comprising 80% of the validation dataset, i.e., the remaining 33.33%-sample of full dataset. For the reduced feature sets, hyperparameter tuning was repeated prior to classifier training and performance evaluation. Balanced accuracy was used as the main parameter to evaluate the classification performance^36^. In addition, the area under the receiver operating characteristics curve (roc-auc)^37^ was calculated. To control possible overfitting, random forests were also trained the reduced feature set with values within variables randomly permuted, with the expectation that a classifier trained with this information should perform no better than guessing, i.e., give a balanced accuracy around 50% and with the 95% cross-validation confidence interval (2.5th to 97.5th percentiles) from the 100-fold cross-validation runs including the 50% guessing level, otherwise overfitting was likely.

#### Generative AI-assisted investigation of outliers in the DAS28-CRP component pattern

With only one or two members in the outlier subgroups (see results section), common oversampling techniques to increase group size were considered insufficient, such as random sampling with replacement that would inadvertently have resulted in identical cases in the training/test/validation data subsets for subsequent supervised analyses or augmented with an arbitrary modification. Generative AI was preferred because it could be based on structural properties of the data set. Therefore, the U-matrix was further extended by computing a P-matrix^14^, which represents the point density in the data space. This density *p(n*_*i*_*)* was estimated as the number of data points in a hypersphere with radius *r* around the prototype vector *w(n*_*i*_*)* for each neuron *n*_*i*_ on the ESOM’s output *grid p(n*_*i*_*)* =*|{data points x| d(w(n*_*i*_*),x)* ≤ *r}|*. The U*-matrix combines distance structures (U-matrix) and density structures (P-matrix) into a single matrix^14^. The ESOM projection is the neighborhood preserving, i.e., data points close to each other in the high-dimensional space are also close to each other on the projection. New data was generated in the neighborhood of a data point (seed) with respect to the distance of the generated point to the seed, which is well defined^38^. The generation uses Bayesian statistics to model the decision of whether a new data point is to be expected, obtaining the probability of the existence of such a data point from the P-matrix, which shows the density of the data of the projection of the data set onto the ESOM. The bandwidth of the density estimate for the P-matrix can be estimated from the distribution of the distances in the U-matrix and is verified in the P-matrix visualization. This was used to generate valid new cases, based on the U-matrix/P-matrix analysis of the observed data. The AI-assisted generation of valid data provided cluster sizes that could be addressed via supervised learning. This allowed the identification of key variables among the DAS28-CRP components that characterized each cluster. Feature selection was used for this task, implemented as a permutation importance calculation in a cross-validation scenario as described above. The classification task was defined as a two-class problem with the cluster of interest versus the other clusters, iteratively through clusters #1,…,#5. Again, feature selection and classifier training were performed in cross-validation scenarios on 2/3 of the dataset, with a 1/3 validation sample separated before the procedure and used only for final validation of the classifiers trained with the full set of d = 4 DAS28-CRP variables, with the selected variables, and for overfitting control, with the permuted selected features.

#### Associations of drug therapy with the severity of DAS28-CRP subscores

Drugs administered to treat PsA were available in the medical record as ATC codes. This was translated into drug classes using information from the DrugBank database^39,40^ at https://go.drugbank.com (version 5.1.10 dated 2023-01-04). The database was downloaded as an extensible markup language (XML) file from https://go.drugbank.com/releases/5-1-10/downloads/all-full-database. The information contained in it was processed using the R package "dbparser"^41^. Drugs coded by ATC number were grouped by drug class using level 1 of the ATC coding specification. Associations with PsA staging and with the magnitude of the DAS28-CRP main subscores, rescaled to quartiles, were analyzed using χ^2^ statistics.

### Ethics approval

The study followed the Declaration of Helsinki and was approved by the Ethics Committee of the Medical Faculty of the Goethe-University, Frankfurt am Main, Germany (19-492_5).

### Consent for publication

All participants provided written informed consent.

## Results

**Figure 2:**
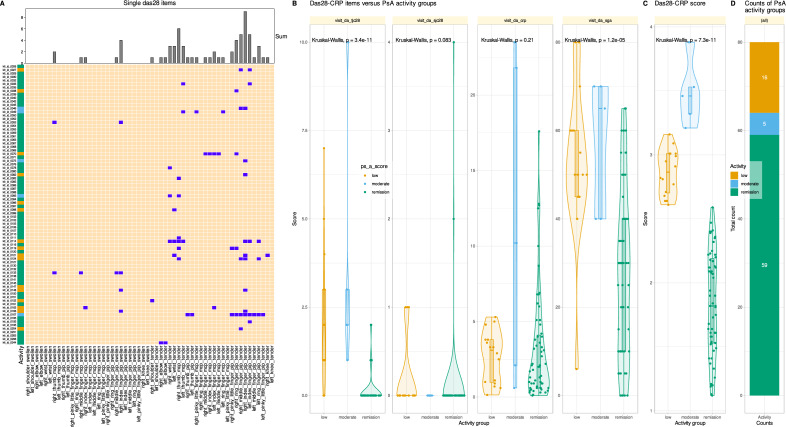
Raw data of the DAS2-CRP score and numbers of patients in grouped for activity of psoriatic arthritis (PsA). (**A**) Matrix plot of tenderness and swollenness of the d = 28 joints queried during scoring. At the top, the marginal sums of affected joints are shown as bar graph. (**B**) Original four components of the DAS28-CRP score and statistical test results. Individual data points are plotted as points on violin plots showing the probability density distribution of the variables, overlaid with box plots where the boxes were constructed using the 25th, 50th, and 75th percentiles of these values. The whiskers add 1.5 times the interquartile range (IQR) to the 75th percentile or subtract 1.5 times the IQR from the 25th percentile. The statistical significance of the differences between PsA activity subgroups for each variable was analyzed using Kruskal–Wallis tests^43^. The p-values obtained are shown above each variable. (**C**) DAS28-CRP sum scores, plotted in a similar manner as the data in panel B. (**D**) Stacked bar chart showing the composition of the PsA patient cohort with respect to disease staging. The figure was created using the R software package (version 4.3 for Linux^8^) and the R libraries "ggplot2"^57^ and "ComplexHeatmap"^58^.

In this report, PsA disease activity stages were graded based on DAS28-CRP grading as defined for its original purpose of evaluating rheumatoid arthritis (< 2.6 remission; ≥ 2.6 to < 3.2: low activity, ≥ 3.2 to 5.1: moderate activity, > 5.1: high activity)^42^. Thus, in the majority of n = 59 PsA patients, psoriatic arthritis was in remission according to the DAS28-CRP, while in n = 16 and n = 5 patients, respectively, it was in low or moderate activity (Fig. 2).

### DAS28-CRP data structures that reflect PsA activity levels

**Figure 3:**
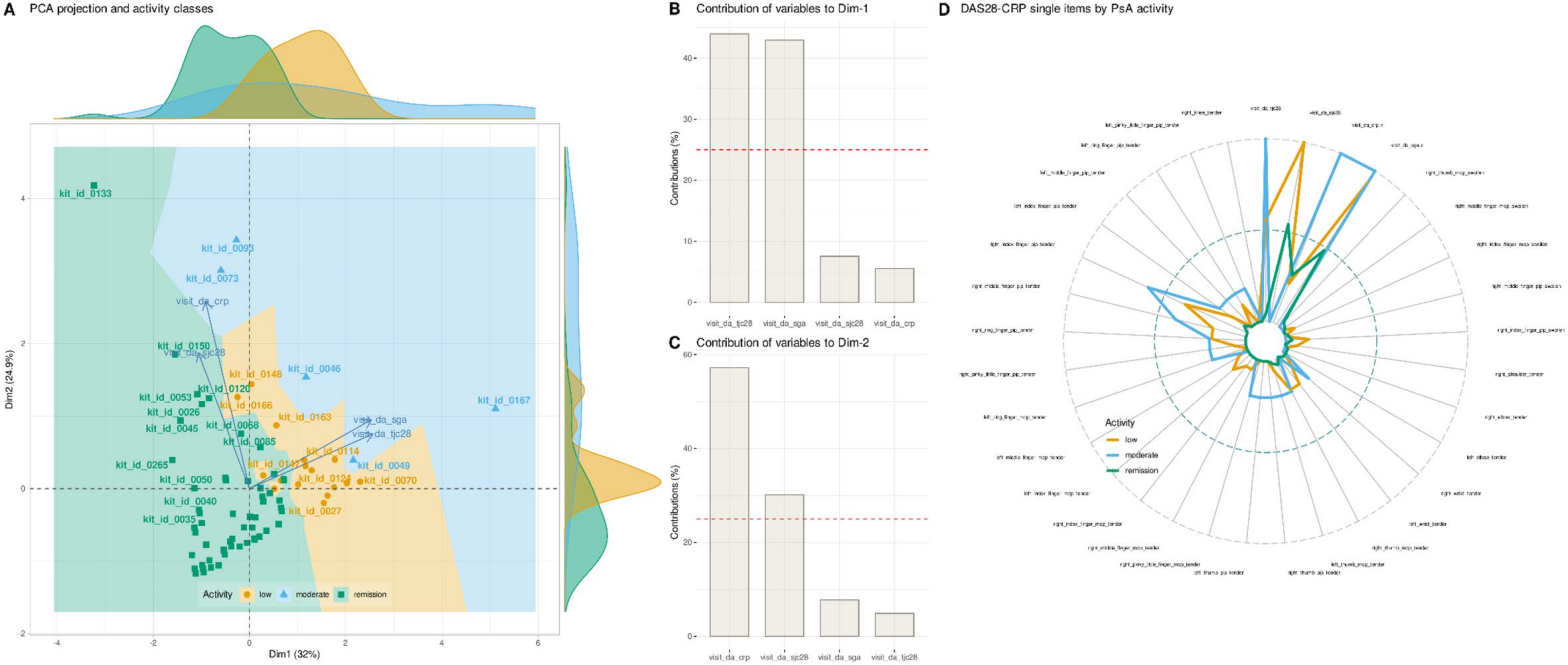
Principal component analysis (PCA) of the original four components of the DAS28-CRP score, i.e., number of tender joints, number of swollen joints, C-reactive protein (CRP), and global health assessment of each individual DAS28-CRP item down to individual joints. (**A**) PCA projection of the data set instances of the original d = 4 DAS28-CRP items. The individual data are represented as points of different colors and shapes according to their membership in the previous classes (patients versus controls). The case coding is shown for reproducibility down to individual patients, depending on available space. The projection plane (dimension 2 vs. dimension 1) consists of Voronoi cells around each data point, colored according to the membership of the respective data point to the previous classes of patients or controls, enhancing the visualization of the data projection as shown recently^59^. (**B**) Bar graph of the contributions of each variable to PC1 and PC2. The dashed horizontal reference line corresponds to the expected value if the contributions were uniform. (**C**) Radar plot of the individual items required to calculate the DAS28-CRP score and the resulting d = 4 score items, separately for each PsA activity subgroup. Data are averaged for previous PsA activity levels. The variables are scaled to the range [0,1]. The figure was created using the R software package (version 4.3 for Linux^8^) and the R libraries “ggplot2”^57^ and “FactoMineR”^12^.

Unsupervised analyses detected structures in the DAS28-CRP that were contingent with the prior classification into disease activity levels. Principal component analysis (PCA) projection of the d = 4 main DAS28-CRP subscores resulted in one principal component (PC) with an eigenvalue > 1 (1.279), while a 2nd PC had an eigenvalue of 0.9943. On the plane created by these first two PCs, patients were segregated by disease activity (Fig. 3 A). The first 2 PCs captured 56.9% of the total variance in the data. The DAS28-CRP items that contributed most to the first PC, along which the separation for PsA activity was most pronounced, were tender joint count (tjc) and swollen joint count (sjc) (Fig. 3 B). This was consistent with the statistical comparisons where the PsA activity subgroups differed significantly only for these variables (Fig. 2). The other two main components of the DAS28-CRP were projected along the 2nd dimension. Breaking the DAS28-CRP down to its individual components (Fig. 3D) showed that the most pronounced symptoms of patients not in remission were, in addition to the main components mentioned, tenderness in the metacarpophalangeal joint of the right index finger (Fig. 3C).

**Figure 4:**
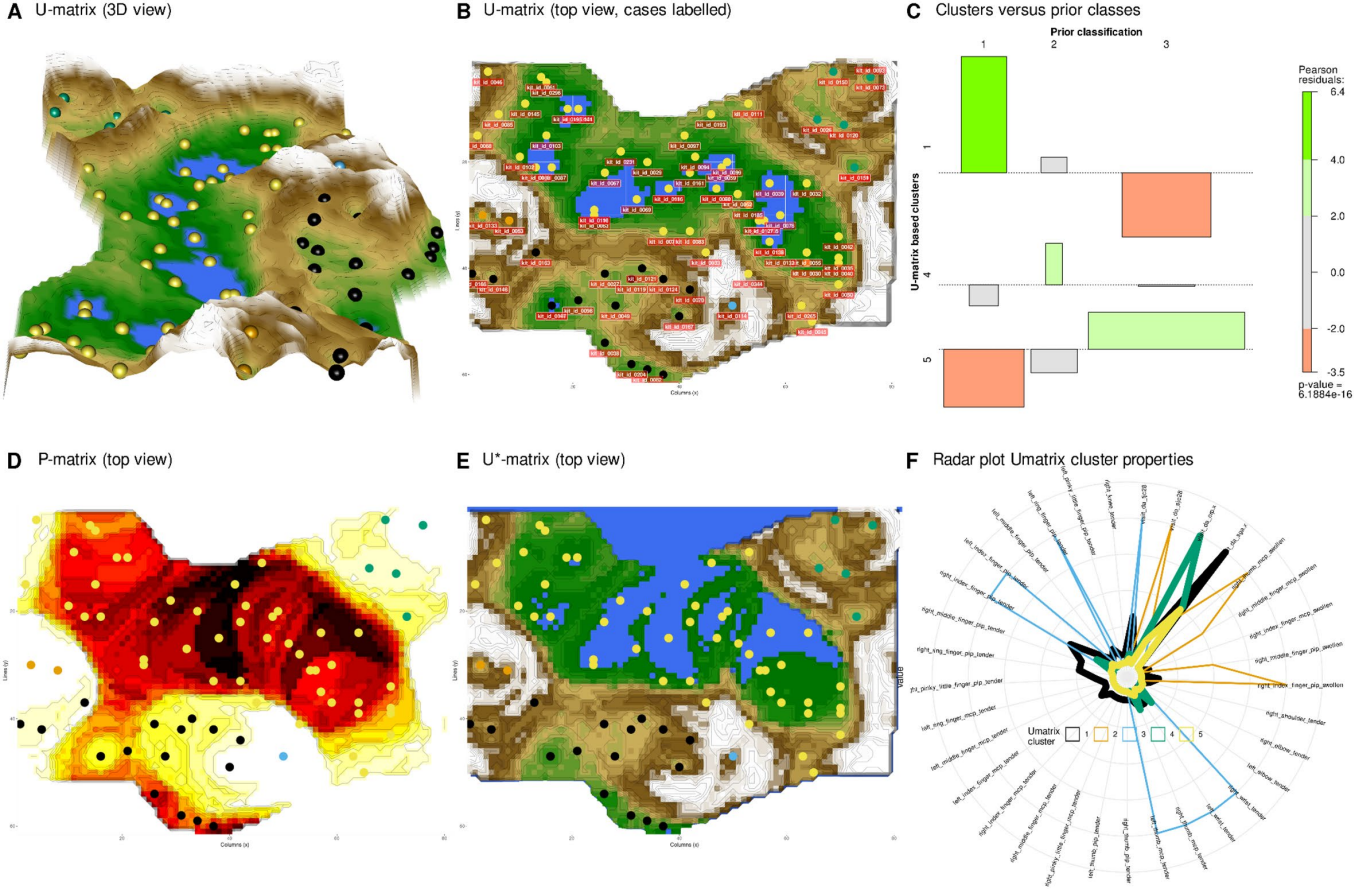
Data structure obtained by SOM-based clustering of the original four components of the DAS28-CRP score, i.e., number of tender joints, number of swollen joints, C-reactive protein (CRP), and global health assessment of each individual DAS28-CRP item down to individual joints. (**A**) Three-dimensional display of the U-matrix visualization of distance-based structures of the DAS28-CRP score. The figure was obtained by projecting the data points onto a toroidal grid of 4,000 neurons (50 × 80) with opposing edges connected. The dots represent the "best matching units" (BMUs) of the self-organizing map (SOM), i.e., the neurons whose weight vector is most similar to the input. A single neuron may be the BMU for more than one data point or subject, so the number of BMUs may not equal the number of measurements acquired. The U-matrix has been colored as a geographical map with brown (up to snow-covered) heights and green valleys with blue lakes. Valleys indicate clusters and watersheds indicate boundaries between different clusters. The BMUs are colored according to the clusters/outliers identified on this basis. (**B**) The top view of the U-matrix is shown in panel A for comparison with the presentations in the following panels. The BMUs are labeled with the patient codes assigned to the respective neuron on the SOM. (**C**) Association plot visualizing the residuals of an independence model for the PsA staging versus clusters contingency table^60^. Clusters with less than 5 members (U-matrix clusters #2 and #3) were not included. The area of each box is proportional to the difference between the observed and expected frequencies. The rectangles in each row are positioned relative to a baseline indicating independence, i.e., if the observed frequency of a cell is greater than the expected, the box rises above the baseline, otherwise it falls below. Each cluster (lines) derived from the U-matrix is plotted against PsA activity level. Prior classes: 1 = low PsA activity, 2 = moderate activity, 3 = remission. (**D**) Data-density based P-matrix with darker colors coding denser data. (**E**) U* matrix resulting from the superposition of the data density-based P matrix with the distance based U matrix to further enhance subgroup separation. (**F**) Radar plot of the individual items required to calculate the DAS28-CRP score and the resulting d = 4 score items, separately for each PsA activity subgroup. Data is averaged for U-Matrix based clusters. The variables are scaled to the range [0,1]. The figure was generated using the R software package (version 4.3 for Linux^8^). Specifically, the U-matrix was plotted using our R package “Umatrix”^15^, and tree and association plots were drawn using the R package “vcd”^20^ including the “strucplot” framework^61^.

Separation for PsA activity was also observed using a separate projection technique as internal validation, i.e., ESOM projection with U matrix, P matrix and their combination, U* matrix (Fig. 4A,B,D,E). There, k = 3 clusters emerged, with sizes n = 17, 7, and 53 cases in clusters #1, #4, and #5, respectively, that were significantly consistent with the prior class structure (χ^2^ = 77.4, df = 4, p = 6.188 × 10^–16^). The association plot (Fig. 4C) showed that in cluster #1, patients with low PsA disease activity were significantly overrepresented while patients in remission were significantly underrepresented. Details of the symptoms pattern are shown in Fig. 4F. The opposite was true for cluster #5, whereas patients with moderate disease activity were overrepresented in cluster #4. In addition, three patients appeared as outliers in the U-matrix, as indicated by their placement in “volcanic craters” on the physical map analogy as the standard representation of this type of SOM. Specifically, two patients (assigned to “cluster” #2) shared one of these localization-indicating outliers, and a third (assigned to “cluster” #3) was completely separated from all others. The unique DAS28-CRP related characteristics of these patients were further explored as reported in a separate paragraph at the end of the results section.

### DAS28-CRP items most relevant for PsA activity staging in peripheral PsA-type

**Figure 5:**
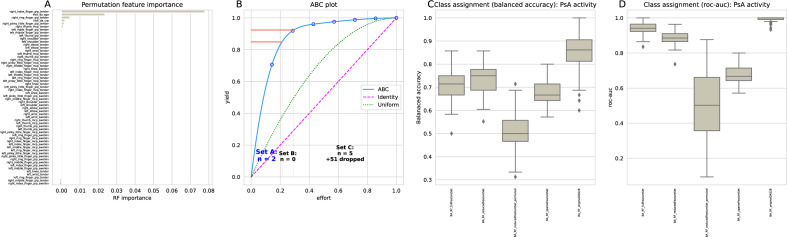
Identification of DAS28-CRP items, among all its d = 58 single items, that were most informative in inferring whether a patient has PsA in remission or not. (**A**) Means of variable importance, calculated as permutation importance, obtained in a 100-fold cross-validated random forest training and testing on the 66.67% training/test sample of the original dataset. (**B**) cABC analysis plot (blue line) showing the cumulative distribution function of variable importance. The red lines show the boundaries between the cABC subsets “A”, “B”, and “C”. Category "A" with d = 2 variables is considered to include the most relevant variables for PsA activity stage discrimination ("reduced feature set”: global health score and presence of tenderness in the metacarpophalangeal joint of the right finger) and only one variable, tenderness in the metacarpophalangeal joint of the right finger. (**C**) Balanced accuracy of class assignment, i.e., whether PsA was in remission or not, of random forest classifiers trained with (i) the full set of d = 58 DAS28-CRP items, (ii) the "reduced feature set" obtained by cABC analysis, (iii) the reduced feature set with all variables randomly permuted, (iv) tenderness in the metacarpophalangeal joint of the right finger only 2 (“sparse feature set"), and (v) the original four components of the DAS28-CRP score, viz, number of tender joints, number of swollen joints, C-reactive protein (CRP), and global health status. Feature selection and classifier training were performed in a 100-fold nested cross-validation setting with subsets randomly drawn from the 66.67% training data subset separated at the beginning of the analyses. The boxes show the 25th, 50th, and 75th percentiles of the balanced accuracy (BA) for the classification performance on the 33.33% validation data, which was separated from the entire dataset before feature selection and classifier training, and was not used for feature selection and algorithm training. The whiskers add 1.5 times the interquartile range (IQR) to the 75th percentile or subtract 1.5 times the IQR from the 25th percentile. (**D**) Roc-auc of class assignment, plotted similarly to the performance measures in panel C. The figure was generated using Python version 3.8.13 for Linux, with the seaborn statistical data visualization package^62^ and our Python package "cABCanalysis" available at https://pypi.org/project/cABCanalysis/.

The supervised analyses focused on the characteristics (features) of PsA patients among the DAS28-CRP items, down to the level of d = 58 individual items, that were most informative for the stages of PsA in terms of disease activity. One finding from the unsupervised analyses was that tenderness at the metacarpophalangeal joint of the right index finger was the most common joint-related symptom in patients not in remission according to DAS28-CRP threshold (Fig. 2). This symptom emerged from the feature selection along with the global health assessment score (SGA), which together provided the "reduced" feature set resulting from the cABC analysis-based item categorization of all DAS28-CRP items with respect to variable permutation importance (Fig. 5).

The two DAS28-CRP items alone were sufficient to train a random forest classifier to detect whether a patient was in PsA remission or not with a balanced accuracy of 75% and an roc-auc of 89% (Table 2), which was better than the classification performance of a classifier trained with all d = 58 individual DAS28-CRP items, i.e., tenderness and swelling in the d = 26 joints, CRP concentration, and general health score, and only slightly below the classification performance of a classifier trained with the four standard DAS28-CRP components containing that contain the sum of affected joints in terms of tenderness and swelling, rather than individual joints marked yes/no (Table 1). Furthermore, tenderness of the metacarpophalangeal joint of the right index finger alone provided sufficient information to train the classifier to assign a new sample, not seen during feature selection and training, to either PsA in remission or not with an accuracy that was safely above guesswork, as indicated by the confidence intervals of the performance measures not including 50% (Fig. 5).

**Table 2:** Performance measures of random forests classifiers for assigning a Patient to PsA activity either in remission or not (mild or moderate activity).

Features	Classification performance	
	Balanced accuracy	roc-auc
Full feature set	0.71 (0.58–0.8)	0.94 (0.88–1)
Reduced feature set	0.75 (0.64–0.86)	0.89 (0.82–0.94)
Reduced feature set permuted	0.5 (0.36–0.67)	0.5 (0.19–0.87)
Sparse feature set	0.67 (0.58–0.75)	0.67 (0.58–0.75)
Original DAS28-CRP	0.86 (0.68–0.94)	1 (0.96–1)

Training was performed on all d = 58 items of the DAS28-CRP score ("full feature set"), on only two items ("reduced feature set": global health score and presence of tenderness in the metacarpophalangeal joint of the right finger), and on only one variable, tenderness in the metacarpophalangeal joint of the right finger. The trained random forests algorithm was then applied to a validation sample consisting of 33.33% of the cases, which had been removed from the dataset in a class-proportional manner prior to feature selection and classifier training and had not been touched until used in the classifier validation task. In addition, the validation task was repeated with permuted training features to observe possible overfitting. For comparison, training was also performed using the original four components of the DAS28-CRP score, i.e., number of tender joints, number of swollen joints, C-reactive protein (CRP), and global health status. Shown are medians and nonparametric 95% confidence intervals (2.5th to 97.5th percentiles) from 4 × 25 nested cross-validation runs.

That is, feature selection and classifier training were performed on the 66.67% training/test subsample of the original dataset, and performance evaluation of the trained classifiers was performed on the 33.33% validation subsample, which was removed at the beginning of the analyses and not touched until the final validation task. As a control for overfitting, the classification accuracy dropped to the guessing level of 50% when training random forests with permuted variables of the “reduced feature” set.

### Associations of systemic drug therapy with disease activity.

**Figure 6:**
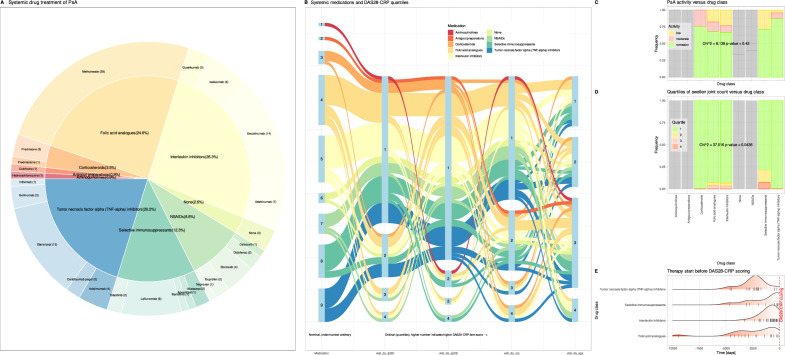
Medications of patients with psoriatic arthritis (PsA), grouped according to level 1 of the ATC drug coding as queried from the DrugBank database^39,40^ at https://go.drugbank.com (version 5.1.10 dated 2023–01-04), and the association of mediation with the four standard items of the DAS28-CRP score. (**A**) Pie chart of drug classes and individual drugs associated with the percentage or number of patients to whom they were administered. (**B**) Sankey plot showing the association of drug classes with a patient’s membership too one of the four quartiles of the observed ranges of the four DAS28-CRP items [1 = low score, …, 4 = high score]. (**C**) Mosaic matrix plot of drug therapy classes versus PsA activity staging. Drug classes administered to only one patient and non-specific drugs are greyed out to increase visual focus. (**D**) Mosaic matrix plot of drug therapy classes versus belonging to one of the quantiles of the swollen joint count subscore. (**E**) Time of therapy initiation from the time of DAS28-CRP score assessment. The plots show the probability density functions (pdf), implemented as the standard kernel density function of the R package "ggplot2"^57^, for treatments used in > 5 patients. The pdfs are shaded between red from the start of therapy long back to white when therapy had just started shortly before the assessment. Individual start points of medication are indicated as small black lines on the x-axes. The figure was generated using the R software package (version 4.3 for Linux^8^) and the libraries “ggforce”^63^ and “ggExtra”^64^.

Details about the substances administered are provided in Fig. 6. Eight different drug classes were administered systemically to the PsA patients, including aminoquinolines (n = 1 patient), antigout agents (n = 1), corticosteroids (n = 4), folic acid analogues (n = 28), interleukin inhibitors (IL-12/23, IL-17 and IL-23 inhibitors specifically) (n = 30), non-steroidal anti-inflammatory drugs (NSAIDs) (n = 10), selective immunosuppressants (n = 14), tumor necrosis factor-alpha (TNF-alpha) inhibitors (n = 23). The start of the medication dated back 9465 – 0 days (median 1243 days; Fig. 6 E). It is worth noting that the drug groups are reported based on the "level_1" grouping in the DrugBank database, and to maintain consistency and reproducibility, no modifications were made. However, it is important to mention that most folic acid analogues administered to patients primarily consisted of the disease-modifying anti-rheumatic drug (DMARD) methotrexate. This fact should be taken into consideration when interpreting the results or examining Fig. 6. Three patients had not yet received specific medications at the time of the DAS28-CRP scoring analyzed for this report. In addition, 13 patients also received topical corticoids, which were not analyzed further.

PsA staging into remission, low or moderate activity did not depend on the class of drug the patient received for PsA therapy (χ^2^ = 8.1384, df = 8, p = 0.4201; Fig. 6C). As expected, there was a tendency that earlier therapy initiation relative to DAS28-CRP assessment was associated with less actual disease activity (Kruskal–Wallis test^43^: chi-square = 5.3085, df = 2, p-value = 0.07035, with median therapy start dates of 554, 134, and 1812 days in patients with actual low, moderate, and remission PsA activity, respectively). A Sankey plot of the drugs versus the scores of the four main components of the DAS28-CRP score, rescaled to quartiles, suggested differential drug efficacy on selected components of the clinical score (Fig. 6B). This was significant for the number of swollen joints (χ^2^ = 37.016, df = 24, p = 0.0436). Patients in the upper quartiles of swollen joint counts were concentrated in the selective immunosuppressive therapy group, which in this cohort included small molecules inhibiting various targets such as Janus kinase, pyrimidine synthesis, phosphodiesterase 4 or cytotoxic T-lymphocyte-associated antigen 4 (Fig. 6D).

#### Generative AI-assisted characterization of outlier patients in the DAS28-CRP component pattern

Three patients emerged as outliers on the ESOM projection of the DAS28-CRP single scores as described above. In order to investigate the DAS28-CRP-related characteristics of these outliers a generative artificial intelligence-based method using artificial neural network-based learning was used. The DAS28-CRP-related characteristics of these outliers were investigated using generative learning neural networks based on a Bayes model of the data’s distances obtained from a valid projection of the data in form of the P-matrix. Based on distance- and density-structure of the data matrix obtained during ESOM projection of the d = 4 DAS28-CRP components (Fig. 4D), the subgroups could be populated with validly generated data to sizes that allowed further exploration of the relevant score items typical of each subgroup. The usable group size was chosen to be n = 53 of the largest cluster #5. All five clusters were enhanced to this size, meaning that 53—17 = 36 generated data were added to the original n = 17 cluster #1, with an analogous addition of n = 51, 52 46 generated cases to clusters #2—4. No generated cases were added to cluster #5. These clusters, now of equal size, could be analyzed for the dominant DAS28-CRP subscore by performing feature selection with random forests.

**Figure 7:**
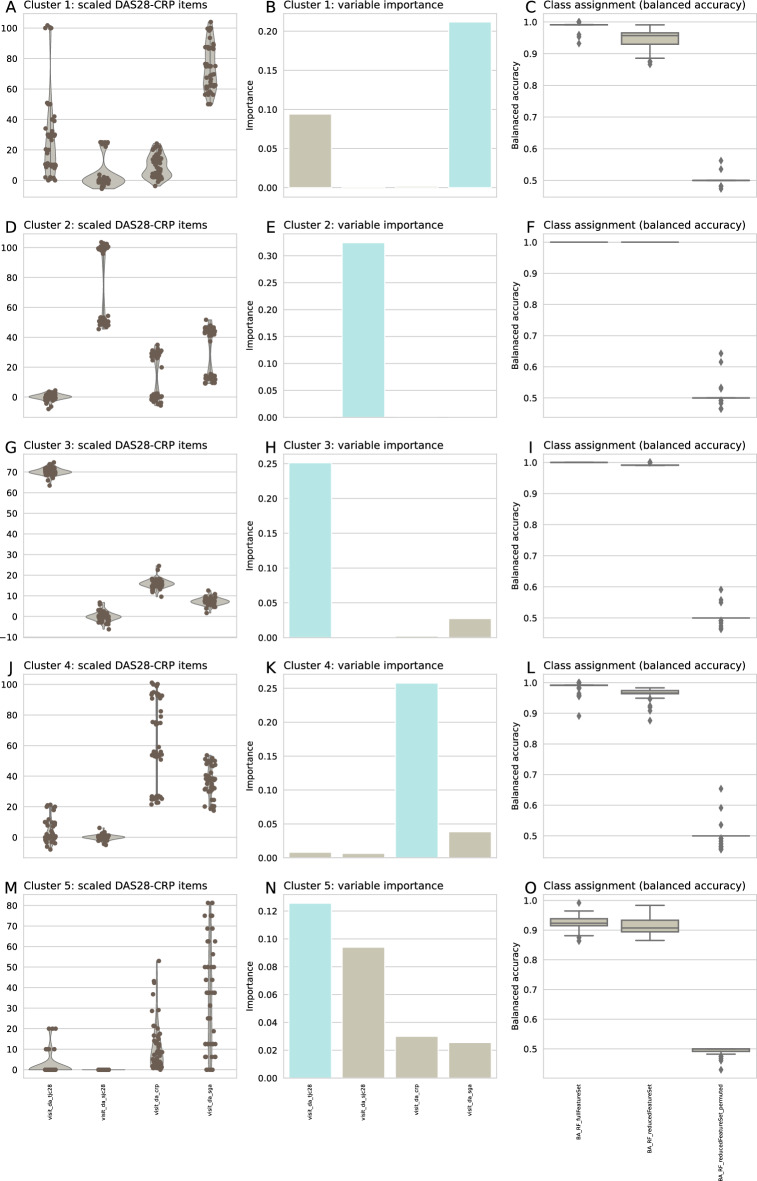
Exploration of the U-matrix derived cluster structure of PsA patients based on the four key components of the DAS28-CRP score, using generative AI to increase the group sizes to an equal size of n = 53 patients in all clusters corresponding to the size of the largest observed cluster. **Left column of panels (A, D, G, J, M):** DAS28-CRP scores, normalized to percent of the observed maximum, separately for each score item and cluster. Individual data points are plotted as points on violin plots showing the probability density distribution of the variables, overlaid with box plots where the boxes were constructed using the 25th, 50th, and 75th percentiles of these values. The whiskers add 1.5 times the interquartile range (IQR) to the 75th percentile or subtract 1.5 times the IQR from the 25th percentile. **Middle column of panels (B, E, H, K, N):** Variables identified by feature selection using the generic permutation importance measure with random forests as the classifier. The bars indicate the mean feature importance in a 100-fold cross-validation experiment using 2/3 of the original data as test/training data subsets with random resampling. The classifiers were trained to discriminate a case's membership in the cluster of interest (#1 to #5 from top to bottom row) from its membership in any other cluster. **Right column of pale (C, F, I, L, O):** Balanced accuracy of cluster assignment of random forest classifiers trained with (i) the full set of d = 4 DAS28-CRP key items, (ii) with one feature identified as most important, and with the same feature with values randomly permuted. The boxes show the 25th, 50th, and 75th percentiles of the Balanced Accuracy (BA) for the classification performance on the 33.33% validation data, which was separated from the entire dataset before feature selection and classifier training and was not used for feature selection and algorithm training. The whiskers add 1.5 times the interquartile range (IQR) to the 75th percentile or subtract 1.5 times the IQR from the 25th percentile. The figure was generated using Python version 3.8.13 for Linux with the seaborn statistical data visualization package^62^.

This showed (Fig. 7) that one of the d = 4 DAS28-CRP score items was sufficient to assign a case to its respective cluster, namely the number of swollen joints was highest in cluster 1 and was sufficient to identify membership to cluster #1 with > 90% balanced accuracy. Cluster #1 contained the most patients with low PsA activity. A low number of tender joints was sufficient to correctly identify membership in cluster #5, which carried most patients in remission. High CRP was characteristic of cluster #4, which overrepresented patients with moderate disease activity. The outliers were patients characterized by either a high number of swollen joints (cluster #3) or a high number of tender joints (cluster #4). The two patients in cluster #3 were both in remission (with DAS28-CRP scores of 1.9 and 2.4) and had unique joint swelling patterns involving the metacarpophalangeal joint of the right thumb and the proximal interphalangeal joint of the right index finger. Treatments varied, with one patient receiving adalimumab and the other receiving a combination of corticosteroids, NSAIDs, and methotrexate. The single patient in cluster #4 had low disease activity (DAS28-CRP = 3.1), joint tenderness in both wrists and metacarpophalangeal joints of both thumbs and was treated with methotrexate and etanercept.

## Discussion

The DAS28-CRP is a score for assessing the activity of peripheral arthritis. While it was originally developed and validated for rheumatoid arthritis, it has been used to assess psoriatic arthritis alongside disease-specific scores such as the Disease Activity in Psoriatic Arthritis Score (DAPSA), which is similarly constructed but includes lower extremity joints, and distal interphalangeal joints, typically affected in PsA but not in RA and a subjective pain assessment^4^. Using information reduction as a typical feature of machine learning, the present analysis showed that among the items collected in a standard clinical scoring tool for psoriatic arthritis, a single joint appears to provide the most relevant information about disease activity. Tenderness in the metacarpophalangeal joint of the right index finger was not only the most common joint-related symptom in the present cohort, but also allowed identification of PsA activity with an accuracy equivalent to that of a machine learning-based classifier trained with all the individual items that make up the DAS28-CRP score. The only other meaningful score item, ranked second after tenderness at the metacarpophalangeal joint of the right index finger, was the patient reported global health status, which was found to be meaningful for the actual stage of PsA activity.

In a disease characterized by skin, nail, and joint manifestations in about 20–30% of patients^1^, joint complaints were expected to play a role in a random sample such as the present cohort. In fact, they were observed more frequently, in n = 25 patients (31.25% of patients with at least one mention of joint complaints), whereas swollen joints were observed in only 5 patients (6.67%). However, the fact that a single joint alone conveyed much of the information was not expected. It must be noted that inflammation in the MCP joints of digits is also a symptom of gout, that is commonly associated with PsA^44^. The frequent comorbidity of the diseases in general could render an independent observation of the symptom difficult^45^. In their initial description of the disease by Moll & Wright five clinical subgroups were defined based on their joint inflammation patterns^46^. These patterns were proposed as “distal interphalangeal joint predominant arthritis (DIP)”, “asymmetrical oligoarticular arthritis”, “symmetrical polyarthritis”, “arthritis mutilans” and “predominant spondylitis”, with asymmetrical oligoarticular arthritis making out a majority of patients^47^. Although not explicitly included in any of these groups, (asymmetric) dactylitis is known to be associated with it^47^ and can be categorized as a form of asymmetrical oligoarticular arthritis^48,49^. The present results emphasize the relative importance of tenderness of the MCP joint of the right index finger among the DAS28-CRP constituents for predicting the PsA disease activity stage with an accuracy of about 70%. These findings suggest that individual constituents of the DAS28-CRP might not be equal in relative importance when reflecting PsA activity. To this adds the observation of outliers who could be characterized by either a high number of swollen joints or a high number of tender joints. This offers a perspective on possible differentiation of subgroups within the group of asymmetric oligoarticular arthritis mentioned above^46,47^, as it does not explicitly mention a distinction between swollen and tender joints. The generative machine learning approach thus offers new possibilities for a more granular stratification of PsA including the characterization of outliers. It should also be noted that components of the DAS28-CRP might not account for other joints relevant for PsA manifestation, as the 28 joints selected by the score were initially defined for the characterization of rheumatoid arthritis. Additional joints (e.g., from TJC68 or SJC66) thus need to be evaluated for improved PsA staging in the future. In addition, the previously criticized lack of DAS28-CRP sensitivity to cutaneous disease manifestations and despite well-known differences in predominantly involved joints compared to RA^5,50,51^. However, the subgroups originally proposed by Moll & Wright in 1973 were formulated using expert judgment at a time when unsupervised data analysis techniques, such as various cluster analysis methods, were limited compared to the range of methods available today. There may be a case for re-evaluating the clinical subgroup structure of PsA; however, this task would be well beyond the scope of the present analysis, and the data set is too small and likely insufficient to cover the necessary full spectrum of PsA subgroups as originally proposed by Moll & Wright.

Consistent with the importance of a joint symptom in PsA staging, joints also provided the most distinctive information about treatment success in the present data set on the day of data collection. While patients treated with different major classes of disease-modifying drugs appeared to do similarly well, the number of swollen joints was greatest in patients treated with drugs of the selective immunosuppressant group according to ATC level 1, i.e., abatacept, apremilast, tofacitinib, baricitinib, and leflunomide. As the pharmacological mechanisms of these agents are heterogeneous^52^, this finding does not allow a general conclusion on the efficacy of drug aiming at specific targets. Conversely, an overrepresentation of these selective immunosuppressants in PsA cases with higher activity may be due to escalation of therapy. This is underlined by several clinical guidelines (e.g., EULAR 2020, GRAPPA 2021, ACR/NPF 2018), which place apremilast in the second line after PsA is refractory to methotrexate or other DMARDs. Similarly, abatacept and tofacitinib (a Janus kinase inhibitor) are recommended only after failure of biologic tumor necrosis factor alpha (TNFα) inhibitors^52,53^.

Generative AI is currently being widely discussed. The present analyses show that its utility extends to small clinical datasets in the context of rheumatology. This required a specific type of generative AI that is able to learn from small data sets, whereas the generative adversarial networks (GAN^54^) on which other types of generative AI are based, such as those used in automated image processing, including medical images of patients with rheumatic diseases^55^, require large training data sets, making them unsuitable for studying the characteristics of outliers, for example. The present analysis included both the assessment of data structures based on the prior clinical classification into PsA activity subgroups from weighted sums of the four DAS28-CRP components and ignoring this established scoring, while looking at the pattern emerging from the DAS28-CRP components without calculating the composite score. Instead, cluster structures on the ESOM were examined. The results indicated that the cluster structures emerging from the unsupervised analysis of the four score components were in principle consistent with the clinical staging derived from the sum scores, which was the expected finding. Generative AI was also consistent with the raw data sets with respect to the observation that patients in remission were characterized by low joint tenderness scores, which was consistent with the analysis of single items pointing to a single finger joint as carrying key information about remission. However, the ESOM cluster-based subgrouping was not completely redundant with the previous clinical staging. Using generative AI, two small subgroups of patients could be investigated, which were particularly characterized by joint problems. In the original dataset, these were outliers and therefore anecdotal. However, generative AI suggested the possibility of rare clinical phenotypic subgroups in PsA.

A limitation of the present study is the relatively small sample size, consisting of 80 PsA patients. Additionally, the distribution of disease activity stages is not uniform, with most patients (approximately 74%) in remission, while low and moderate activity cases account for around 20% and 6% of the cohort, respectively. This imbalance is largely attributed to therapeutic management aimed at controlling disease activity. Nonetheless, it is important to note that the issue of imbalanced group sizes has been addressed in all data analyses, for example by employing balanced accuracy as a performance measure for classifiers. While a similar distribution of PsA severity might arise in larger cohorts, validation of the present findings on a larger sample is essential. Furthermore, the present study primarily focused on the DAS28-CRP scoring system, with specific attention given to the relative importance of its individual components down to the single joint level. A comparative analysis across various scoring systems for PsA was not explored, such as DAPSA or PSARC. As mentioned in the introduction, previous research indicated a high correlation between DAS28-CRP and PsA-specific indices^5^. Nevertheless, future research endeavors may delve into similar analysis procedures and analogous findings within competing scoring systems, providing a more comprehensive understanding of PsA assessment.

## Conclusions

Out of the 58 individual items that make up the DAS28-CRP score, the most informative indicator of whether PsA was in remission or not was tenderness in a single joint, followed by the patients' general health self-rating. These results support the previously suggested re-evaluation of DAS28-CRP in PsA^56^, for which this report provides specific guidance on relevant items to focus on. The decomposition of the DAS28-CRP score into its components allowed the identification of informative symptoms of PsA activity. However, relying solely on the final score to determine clinical grading, which is a weighted sum of the four major components of DAS28-CRP, implies a logical "OR" with respect to the score components, and may not capture all relevant subgroups with specific symptoms. This is, however, the standard approach of evidence-based medicine where statistical group effects are sought. Focusing on individual scores using generative AI, on the other hand, can enable precision medicine by targeting small subgroups and addressing outliers. Therefore, the present analysis suggests the need for specific diagnostic or therapeutic approaches that target subgroups with specific symptoms.

## Data Availability

The data sets generated and analyzed in the current study are not publicly available. The data are available from the first author upon reasonable request and on approval by our ethics committee.
